# Exclusion criteria and generalizability in bipolar disorder treatment trials

**DOI:** 10.1016/j.conctc.2018.01.009

**Published:** 2018-01-31

**Authors:** Jessie J. Wong, Nev Jones, Christine Timko, Keith Humphreys

**Affiliations:** a Center for Innovation to Implementation, VA Palo Alto Health Care System, Palo Alto, CA, USA; b Center for Health Policy/ Center for Primary Care and Outcomes Research, Stanford University, Stanford, CA, USA; c Department of Mental Health Law & Policy, University of South Florida, Tampa, FL, USA; d Department of Psychiatry and Behavioral Sciences, Stanford University, Stanford, CA, USA

**Keywords:** Generalizability, Bipolar treatment, Research design, External validity, Translation

## Abstract

**Objective:**

The current paper reviews the English-language research on exclusion criteria in bipolar disorder treatment trials and discusses how study samples compare to the general bipolar patient population.

**Methods:**

& Results: Across 8 identified studies of exclusion criteria and their impact, between 55% and 96% of people with bipolar disorder would be excluded from treatment research. The number of exclusion criteria varies across bipolar disorder treatment research, with one study estimate of a median of 7 criteria used across studies. The criteria that excluded the greatest number of potential participants were comorbid substance use disorder, suicidal risk, and comorbid medical conditions. Both studies that compared treatment responses among participants who met and did not meet exclusion criteria found no statistically significant differences.

**Conclusions:**

Most potential participants are excluded from outcome research, which creates challenges for recruitment and limits generalizability of study findings. Common exclusionary practices lead to unrepresentative samples that limit generalizability and reduce the confidence of clinicians that findings can be translated to front-line practice with bipolar disorder patients.

## 1 Introduction

Bipolar disorder affects 4.4% of the population at some point in their lifespan [1] and often causes significant disruptions to work, social, and family life domains [2] as well as increased suicidal risk [3]. The nature of bipolar disorder and its comorbidities present unique challenges to treatment researchers, including how to select exclusion criteria that balance rigor and relevance [4]. More stringent exclusion criteria can increase the likelihood that a sample will respond to an evaluated treatment in a homogeneous fashion, which enhances statistical power. Yet, exclusion criteria by definition widen the gap between research samples and clinical populations, thereby threatening external validity.

Clinical trials across a range of psychiatric disorders have traditionally attempted to recruit samples of individuals with symptoms (and related impairment) that emanate exclusively from their primary diagnosis [5]. This approach to sample selection has raised concerns regarding the generalizability of research samples to ‘real-world’ community patient populations, most particularly whether “evidence-based” treatments are effective for the severely troubled patients who tend to be excluded from clinical trials [6]. Some exclusion criteria are essential to treatment research in order to protect human subjects from potential harm (e.g., adverse medication interactions). Yet others are optional and, as such, it is important to consider how the exclusion criteria may influence study samples and the implications of potentially biased samples regarding the generalizability of treatment effects. This paper reviews the literature on the exclusion criteria that have been employed in bipolar disorder treatment research, the proportion of patients excluded, and how exclusion criteria may affect the generalizability of results.

## 2 Methods

The Cross-disease Review of Exclusion Across Medicine (CREAM) project is assessing the impact of exclusion criteria in research conducted across a range of medical specialties (e.g., psychiatry, oncology, rheumatology). A detailed description of the literature review procedure can be found in Humphreys [7]. Literature was primarily identified by conducting English-language searches in PubMed (Original Date of Search: July 8, 2013) on the following terms: ‘Eligibility criteria and generalizability’ (anywhere in paper), ‘exclusion criteria and generalizability’ (anywhere in paper), ‘exclusion criteria’ (in title of paper) and ‘eligibility criteria’ (in title of paper). This generated 326 unique articles, all of which were reviewed by one of the authors, as were relevant references within those articles. An updated search was conducted on August 19, 2016, which yielded an additional 160 unique articles. Other articles were discovered in a frankly opportunistic fashion. From this cross-disease pool of literature, evidence related to specific diseases were synthesized within focused reviews for a range of diseases, including depression [8], neurological diseases [9], schizophrenia [7], anxiety disorders [10], and substance use disorders [11]. The present review focuses on those identified treatment studies that address overall and/or specific criteria exclusion rates for bipolar disorder.

To be considered relevant to the CREAM project, studies had to analyze data on (1) the prevalence and nature of exclusion criteria, and/or (2) the impact of exclusion criteria on sample representativeness or study results. Clinical trials that simply reported their exclusion rate were not included in this review. Non-participation in research based on lack of informed consent by eligible participants has quite different implications than exclusion based on factors selected by the researchers. For this reason, lack of informed consent was not considered as an exclusion criterion in the current review.

## 3 Results

All included studies (*N* = 8) evaluated baseline differences between included and excluded participants in bipolar disorder treatment studies and two studies also examined outcome differences. Major findings are summarized in Table 1. Potential subjects excluded based on specific exclusion criteria within each study is presented in Table 2.

**Table 1:** Summary of study finding.

First author (Year)	Sample size and characteristics	Criteria that excluded the most potential participants (exclusion rate by criterion)	Exclusion rate
Licht (1997)	164 inpatients with manic symptoms	1) “Methodological reasons” (39%) 2) “Non-compliance” (32%) 3) “Safety reasons” (4%)	84%
Talamo (2008)	67 bipolar, acutely manic inpatients	1) Comorbid substance use (52%) 2) Recent suicide attempts (38%) 3) Violent acts (23%)	78%
Zarin (2005)	92 patients with acute mania currently receiving psychiatric services	1) Comorbid substance use (39%) 2) Uncontrolled major medical disorders (22%) 3) Central nervous system/neuromuscular disorder (6%)	55%
Sachs (2012)	504 bipolar patients with manic or mixed episode from across 47 centers	Individual criteria not examined.	64%
Hoertel (2013)	785 bipolar depression and 724 bipolar mania community dwelling patients	1) Comorbid substance use (36%, 36%) 2) Suicide risk (24%, 21%) 3) Comorbid medical condition (20%, 19%)	58%, 56%
Bowden (1995)	179 participants and 577 potential participants with acute mania	1) Failure to meet diagnostic criteria (32%)	Not available
Zimmerman (2016)	Not available (Analysis conducted at study-level; 22 studies)	1) Depressive symptom severity 2) Suicidal ideation 3) Alcohol/drug use disorder 4) Comorbid psychiatric disorder 5) Duration of current depression episode 6) Current manic symptoms	Not available
Filkowski (2015)	163 treatment-refractory bipolar patients referred from physicians, self, or family	1) Psychiatric comorbidity (48%) 2) No prior ECT (32%) 3) Not meeting minimum severity requirements (21%)	96%

Note. The Filkowski et al. (2015) study was unique in studying deep brain stimulation. The results from this study may not generalize to studies of other treatment types.

**Table 2:** Percentage of potential study participants excluded from studies based on specific exclusion criteria by study.

First Author (Year)	Licht (1997)	Talamo (2008)	Zarin (2005)	Sachs (2012)	Hoertel (2013)	Bowden (1995)	Filkowski (2015)
Overall exclusion rate	84%	78%	55%	64%	58%, 56%	–	96%
Comorbid substance use	–	52%	39%	–	36%, 36%	–	–
Suicide risk/recent suicide attempts	–	38%		–	24%, 21%	–	2%
Comorbid medical condition	–	–	22%	–	20%, 19%	–	17%
Age (<18 or >65)	–	–	–	–	–	–	2%
Psychotic features	–	–	–	–	6%, 5%	–	
Psychiatric comorbidity	–	–	–	–	–	–	48%
Anxiety disorder	–	19%		–	–	–	
Failure to meet diagnostic criteria/severity requirements	–	–	–	60%	–	32%	21%
Violent Acts	–	23%	–	–	–	–	–
CNS/neuromuscular disorder	–	–	6%	–	–	–	–
Pregnancy/lactation	–	–	–	–	7%	–	–
Failure to meet diagnostic criteria based on computerized assessment	–	–	–	15%	–	–	–
Improvement in symptoms from screening to baseline	–	–	–	19%	–	–	–
No Prior ECT	–	–	–	–	–	–	32%
Contraindication	–	–	–	–	–	–	12%
“Safety reasons” (e.g., comorbid substance use, pregnancy, medical conditions)	4%	–	–	–	–	–	–
“Methodological reasons”	39%	–	–	–	–	–	–
“Non-Compliance”	32%	–	–	–	–	–	–

*Note.* --- indicates that this figure was not reported within the corresponding article. Hoertel et al. (2013) results presented for bipolar depression, bipolar mania. Zimmerman et al. (2016) omitted as this level of data was not reported.

Licht and colleagues [12] examined a sample of 164 prospective participants who were deemed eligible for a research study based on initial screenings, comparing those who were subsequently included versus excluded based on various criteria. This sample was drawn from inpatients with manic symptoms who had been consecutively admitted to a university hospital. Thirty-nine percent of the sample was excluded for “methodological reasons” (defined as receiving treatment up to the time of admission), and another 32% were excluded based on “non-compliance.” Another 4% were excluded for “safety reasons,” which encompassed having a major medical illness, being pregnant, known contraindications to the pharmacological treatment of interest, and/or being in an “extreme manic state requiring other treatment.” The combined effect of the criteria was to exclude 84% of the participants who had already been deemed eligible during initial screenings.

Talamo, Baldessarini, and Centorrino [13] reviewed 21 randomized clinical trials (RCTs) conducted between 1998 and 2008 and identified 16 exclusion criteria that were widely-used, non-overlapping and which they had the data to operationalize in medical records of a sample of 67 bipolar inpatients who had received antipsychotic, antimanic, or mood-stabilizing medicines and had a diagnosis of mania or a mixed manic-depressive state at the time of discharge. Medical record data were most commonly excluded based on patients' comorbid substance use (52%) and a recent suicide attempt (38%). A total of 78% of potential participants' records were excluded by at least one criterion, which is a conservative estimate of what would be obtained in real-world patient samples because Talamo et al.’s sample had already been subjected to some exclusion criteria before being selected for analysis. No statistically significant differences were found between excluded and included patient records based on several demographic variables (including age, gender, marital status, employment, education), illness history, current clinical presentations, or treatment outcome. Regarding treatment received, however, the excluded patients were 24% more likely to receive 2 or more psychotropic agents at discharge, which may have influenced their treatment outcome. Data only captured a constrained period of time (11–13 days), which may have also limited ability to detect differences.

Zarin, Young, and West [14] identified a set of 3 exclusion criteria based on 2 published RCTs for valproate and applied them to a sample of DSM-IV diagnosed bipolar disorder patients (*N* = 92) drawn from routine psychiatric practice. All patients in these samples were receiving psychiatric services at the time of data collection. A total of 39% of the sample would have been excluded for a substance use disorder diagnosis, 22% for uncontrolled major medical conditions, and 6% for central nervous system/neuromuscular disorders. Despite only 3 criteria being evaluated, 55% of the sample was excluded under at least one criterion.

Sachs et al. [15] used a sample of 504 potential participants with a primary diagnosis of bipolar disorder, manic or mixed episode from across 47 centers to compare signal detection based on diagnostic criteria as applied by either clinical raters or a computerized assessment. About two-thirds (60%) of the individuals who were excluded based on computerized assessment did not meet the key protocol-specified eligibility requirements related to diagnosis and symptom severity at both screening and baseline. Approximately 19% of the potential sample exhibited improvements in their bipolar symptoms during the elapsed time between screening and baseline interview, which made them ineligible. All the participants identified as ineligible based on the computerized assessment results were also deemed ineligible by clinical raters. Conversely, only 64% of individuals who were considered ineligible based on clinical raters would have also been deemed ineligible by the computerized assessment, suggesting that clinical raters apply exclusion criteria more stringently than computerized programs designed for the same purpose. Regarding treatment response, there were no statistically significant differences between treatment conditions and placebo for any group, although the authors reported an overall trend of greater response to treatment among individuals who would have been included versus those excluded. These results should be interpreted with caution, however, given that the lack of significance may be due to either limited effect size or low power.

Hoertel and colleagues [16] identified exclusion criteria used by at least 10% of a total sample of 32 randomized controlled trials (RCTs) for bipolar depression and 55 for acute bipolar mania. The median number of exclusion criteria was 7, and the researchers had data available to evaluate 6: comorbid substance use disorder, major medical conditions, psychotic features, suicidal risk, pregnancy or lactation, and current use of any psychotropic medication. The operationalized criteria were applied to a sample of community-dwelling individuals with bipolar depression (*N* = 785) and bipolar mania (*N* = 724) drawn from 2001 to 2002 National Epidemiologic Survey on Alcohol and Related Conditions data. Of note, these diagnoses were made based on a fully structured diagnostic interview. Comorbid substance use disorder (36% for bipolar depression; 36% for bipolar mania), suicide risk (24% for bipolar depression; 21% for bipolar mania), and a major medical condition (20% for bipolar depression; 19% for bipolar mania) were the criteria most likely to exclude individuals from enrolling in a treatment research study. The majority (58%) of individuals with bipolar depression and 56% of individuals with bipolar mania would have been excluded from the study based on at least one exclusion criterion. The expected exclusion rate was even higher (64%) within the subsample of 276 individuals seeking bipolar depression treatment.

Bowden et al. [17] compared descriptive statistics between 179 clinical trial participants and 577 prospective participants screened for another clinical pharmacotherapy trial for acute mania. The authors estimated that more than 10 patients with bipolar disorder would have to be screened to enroll 1 patient in an RCT. Comparison between individuals who were included in clinical trials and those who were drawn from epidemiological samples showed similarities across baseline characteristics.

Zimmerman and colleagues [18] recently identified 6 inclusion/exclusion criteria that were used in the majority of a sample of 22 bipolar disorder treatment efficacy trials: minimum severity of depressive symptoms, suicidal ideation, alcohol/drug use disorder diagnosis, comorbid Axis I disorder, length of current depression episode, and current manic symptoms. Similar to treatment studies of major depressive disorder, bipolar treatment studies frequently excluded patients based on comorbid psychiatric and substance use disorders and insufficient severity of depressive symptoms.

Filkowski and colleagues [19] assessed exclusion within a RCT of deep brain stimulation. This study is unique in the literature because of its focus on deep brain stimulation treatment as well as its sample consisting of bipolar patients who were treatment-refractory to pharmacological and psychological treatments. Potential participants were recruited via physician, self, or family referral and underwent phone screenings prior to determination for inclusion/exclusion. Most potential participants with bipolar disorder were excluded for comorbid psychiatric diagnoses (48%) and lack of prior electroconvulsive therapy (ECT; 32%), followed by not meeting minimum symptom severity criteria (21%). This study also reported the highest overall exclusion rate of 96%, leaving only 7 eligible patients out of their initial pool of 163 potential participants. Due to the nature of this treatment, recruitment was likely more difficult and exclusion efforts were likely more rigorous than for drug trials. For these reasons, the results of this study may not generalize to studies of other treatment types.

## 4 Discussion

The current review of exclusion criteria in clinical trials for bipolar disorder treatment found total exclusion rates that ranged from 55% to 96% of potential participants. The 55% rate calculated by Zarin and colleagues is likely an understatement given that 7 was the median number of exclusion criteria employed used across studies and Zarin et al. only evaluated 3 criteria. Hoertel et al. [17] also found exclusion relatively lower exclusion rates at 58% and 56% of individuals with bipolar disorder in the community who may not have been receiving treatment at the time, but the rate was higher (64%) among those individuals who were currently seeking treatment and therefore better represent the typical sampling pool in a clinical trial. At the other extreme of the observed range, Filkowski and colleagues' reported exclusion rate (96%) may be attributed to their exclusion criterion of not receiving “adequate electroconvulsive therapy in the past,” which excluded the second largest proportion of their sample and related to a unique pre-requisite of their specific treatment modality of interest (deep brain stimulation). The remaining studies reported a range of exclusion between 64% and 84% of potential participants with bipolar disorder. To avoid false precision, we would characterize this as an average exclusion ratio in the field of about 3 of every 4 bipolar disorder patients being ineligible for treatment research. This figure is similar to that found for other psychiatric disorders [5].

Exclusion criteria in bipolar treatment trials are sometimes necessary and useful, but can also make recruitment more difficult and restrict the generalizability of study findings. Including more representative bipolar patients in research samples could reduce recruitment barriers and broaden applicability of studying findings across bipolar patient populations. Although the studies in the current review did not provide rationales for their choices regarding exclusion criteria, other than use in previous clinical trials, explicit justification for criteria are critical to assessing the need for and impact of each criterion. Understanding the implications of exclusion in bipolar treatment trials is particularly pertinent given the complexities of bipolar disorder symptoms and the fact that bipolar patients were traditionally excluded from antidepressant trials due to researchers' fears that treatment might induce a manic episode [[20], [21], [22]]. Even when a study's exclusion criteria are necessary for ethical reasons, it remains important to understand how these criteria may influence study implications and generalizability.

Remarkably, although exclusion criteria are often applied in the hopes that they will reduce heterogeneity in treatment response, we found no evidence that they have this effect in bipolar disorder treatment research. Indeed, both studies reviewed found that excluded and included patients did not significantly differ in their outcomes, suggesting that criteria may not have the benefit that putatively trades off their downsides (e.g., increased time and resources needed to recruit a sample, poorer generalizability). Nevertheless, further research in this area with reported effect sizes are necessary to verify the results from these two studies.

The four criteria that accounted for the greatest proportion of exclusion across studies were comorbid substance use disorder, suicide risk, major medical disorders, and minimum symptom severity. Excluding bipolar patients on these bases has important implications for generalizability. To take the example of substance use disorder, very high comorbidity rates have been found among clinical samples of bipolar I (61%) and II patients (48%) [23,24]. Researchers may avoid including patients with this comorbidity, particularly given evidence that suggests comorbid substance use disorders are inversely associated with medication treatment compliance [25] and substance use disorder is related to poorer treatment response among bipolar patients [26], [27]. However, explicit statements based on CONSORT guidelines that clarify the intention and rationale for all exclusion criteria, including comorbid substance use disorder, should be encouraged, as should statements in discussion sections that remind readers of the limits on generalization produced by any exclusion criteria employed [28].

Exclusion for suicidal risk also has a significant impact on external validity. Suicidality is over 20 times higher among patients with bipolar disorder compared to the general population [3]. Although treatment research may exclude suicidal patients out of fear of possible increases in suicidal risk as a result of treatment administration, suicidal risk is present across patients and treatment studies regardless of who is excluded, warranting appropriate supervision and safeguards within all study designs. Further, the monitoring for suicide risk that is employed within research studies is often more comprehensive than what is traditionally provided in real-world clinical settings, suggesting that including patients at suicidal risk in clinical trials may actually be more ethical than excluding them.

Exclusion on the basis of medical comorbidities also affects external validity because of their prevalence among bipolar patients [29,30]. One study estimated 32% of adults with bipolar I disorder have one or more general medical condition [31]. Other estimates range for 15% for thyroid disease to 46% for neurological conditions [24]. Thus, significant and representative portions of the bipolar patient population will be excluded from treatment research.

Several of the identified study findings suggest that not meeting sufficient diagnostic or severity criteria is a particular issue within bipolar disorder treatment research [15,17,19]. Filkowski, Mayberg, and Holtzheimer explain that fluctuations in mood states, which are inherent in bipolar disorder, cause the minimum duration of current episode criterion to be difficult for potential participants to meet, especially across various time points (i.e., screening, baseline interview). Some research has demonstrated that baseline bipolar disorder severity is positively associated with number of psychiatric comorbidities, which is one of the most common exclusion criteria [32]. As such, studies may be employing exclusion criteria (i.e., minimum severity level, no comorbidity) that conflict with one another. Clinical researchers may want to re-evaluate such severity criteria in order to ensure that they are not inadvertently excluding patients based on the very characteristics of the disorder that they are attempting to treat.

Our review also found that the populations from which potential participants are recruited also influence exclusion rates. Studies recruiting from inpatient hospitalizations had exclusion rates around 80% whereas samples recruited primarily from outpatient or community settings ranged from 55 to 65%. As expected, comorbidities and suicidality rates were found to be much higher among inpatient/hospitalized samples than community-dwelling and nationally representative samples. For instance, comorbid substance use was found in 36% of Hoertel's potential pool of community-dwelling individuals, whereas Talamo, Baldessarini, and Centorrino's sample of inpatients included 52% with comorbid substance use. Thus, limits to generalizability due to exclusion criteria are particularly relevant among studies that focus on treatments that are administered to inpatient and/or populations of hospitalized bipolar patients.

### 4.1 Limitations

Unpublished reports, reports that used alternative terminology, and reports that were not referenced in PubMed may have been overlooked by the CREAM projects review method. In addition, lack of reporting on recruitment strategies and/or use of informal selection criteria in many of the studies reviewed limited our ability to identify potential other exclusion criteria and/or biases related to self-selection that may impact the samples that were examined.

### 4.2 Future directions

The current review sought to inform future decisions about exclusion criteria in studies of treatment for bipolar disorder. In a typical bipolar disorder treatment study, about 3 in 4 people with the disorder will be excluded from participation. This raises justifiable concern among clinicians about whether the findings of research can be safely and confidently applied in front-line clinical practice. The revised CONSORT statement requires reporting of exclusion criteria, which should increase the likelihood that readers will be able to understand the population to which the results apply. In addition, medical research often fails to provide explicit rationales for the use of each exclusion criterion [33]. We suggest that researchers make their rationale for exclusion criteria explicit. Specific to studies of bipolar treatments, we recommend that future studies make a concerted effort to assess and report the current phase of illness (e.g., mania, hypomania, depression) among their bipolar patients at the time of recruitment and treatment administration. Such data provide critical information about the timing and context for the efficacy of bipolar disorder treatments. We also encourage researchers designing clinical trials to recruit large, inclusive bipolar samples from the general population, in order to stratify samples based on common exclusion factors to examine these factors as potential moderators of treatment effects and identify subgroups for whom treatments are particularly effective or ineffective. We hope that this review will open a conversation about whether relaxing exclusion criteria in clinical research on bipolar disorder treatment could increase the generalizability and clinical relevance of findings, particularly for the most vulnerable patients in the health care system.

## Ethics approval and consent to participate

Not applicable.

## Consent for publication

Not applicable.

## Availability of data and materials

Not applicable.

## Conflicts of interest

There are no competing interests.

## Funding

Dr. Wong's work was supported by the Department of Veterans Affairs (VA) Office of Academic Affiliations Advanced Fellowship in Health Services Research and Development (HSR&D) and the VA HSR&D Service. Dr. Humphreys' work was supported by a VA HSR&D Research Career Scientist Award (RCS 04-141). Dr. Timko was supported by the VA Health Services Research and Development (HSR&D) Service (RCS 00-001). The sponsor had no role in the design or analysis of this study.

## Appendix A Supplementary data

mmc1 mmc1
